# Hysterectomy and women’s health in India: evidence from a nationally representative, cross-sectional survey of older women

**DOI:** 10.1186/s40695-022-00084-9

**Published:** 2023-01-06

**Authors:** Sapna Desai, Roopal Jyoti Singh, Dipti Govil, Devaki Nambiar, Ankita Shukla, Hemali Heidi Sinha, Rajani Ved, Neerja Bhatla, Gita D. Mishra

**Affiliations:** 1 Population Council Institute, Zone 5A India Habitat Centre, Lodhi Road, New Delhi, 110003 India; 2 International Institute for Population Sciences, Mumbai, India; 3 George Institute for Global Health, New Delhi, India; 4 Independent Consultant, New Delhi, India; 5 Department of Obstetrics and Gynaecology, All India Institute of Medical Sciences, Patna, India; 6 Bill & Melinda Gates Foundation, New Delhi, India; 7 Department of Obstetrics and Gynaecology, All India Institute of Medical Sciences, New Delhi, India; 8 University of Queensland School of Public Health, Herston, Queensland Australia

**Keywords:** Hysterectomy, India, Chronic disease, Women’s health, Menopause

## Abstract

**Background:**

Hysterectomy, particularly when conducted in women younger than 45 years, has been associated with increased risk of non-communicable diseases. In India, research indicates that hysterectomy is a common procedure for women, but there have been no studies on its long-term effects. We examined patterns of hysterectomy amongst women in India and associations with their health and well-being in later life.

**Methods:**

This analysis utilised the first wave of the Longitudinal Study on Aging in India, a nationally representative study of adults that included a module on health and well-being.

We analysed data on 35,083 women ≥45 years in India. We estimated prevalence of hysterectomy and performed multivariable logistic regression to identify associated risk factors and to examine the association between hysterectomy status and eight self-reported chronic conditions, hospitalisation and mobility.

**Results:**

The prevalence of hysterectomy among women >=45 years was 11.4 (95% CI: 10.3, 12.6), with higher odds among urban women (aOR: 1.39; 1.17,1.64) and higher economic status (highest compared to lowest quintile: aOR: 1.95; 1.44, 2.63). Hysterectomy history was associated with four chronic conditions: hypertension (aOR: 1.51; 95% CI: 1.28, 1.79), high cholesterol (aOR: 1.43; 1.04, 1.97), diabetes (aOR: 1.69; 1.28, 2.24), and bone/joint disease (aOR: 1.54; 1.20, 1.97) and higher odds of any hospitalisation in the past year (aOR: 1.69; 1.36, 2.09).

**Conclusions:**

In India, evidence suggests that hysterectomy is associated with major chronic conditions. The assessment for hysterectomy as a treatment option for gynaecological morbidity should consider potential health consequences in later life.

**Supplementary Information:**

The online version contains supplementary material available at 10.1186/s40695-022-00084-9.

## Introduction

Hysterectomy, removal of the uterus, is a common surgical procedure used to treat gynaecological morbidities such as fibroids, cysts, and uterine prolapse in women typically close to or after menopause [1]. Hysterectomy prevalence varies widely across high-income countries, ranging from 173/100,000 women in Denmark to 510/100,000 in the United States, with many countries reporting a decline over time due to advances in alternative interventions [1]. Hysterectomy accompanied by removal of the ovaries (oophorectomy) surgically induces menopause, while uterine removal alone is associated with decline in ovarian function [2–4]. A 2022 systematic review of 29 studies on the long term effects of hysterectomy –both with and without oophorectomy—indicated evidence of an association between hysterectomy and chronic diseases among women, including an increased risk of cardiovascular events, cancers, depression, metabolic disorders, and dementia [5]. Risks are higher for women whose ovaries were concurrently removed, due to loss of oestrogen. However, emerging evidence from cohort studies indicates that hysterectomy with ovarian preservation is associated with higher risk of cardiovascular disease and metabolic disorders [6, 7] and all-cause mortality for women who underwent hysterectomy before age 50 and without hormonal therapy. As a result,the common use of hysterectomy is a critical issue for women’s health through the life course, both as a reflection of inequitable access to health services and for its long-term consequences for women’s health [8].

Population-based research on hysterectomy has largely focused on high-income settings, with limited understanding of the prevalence, risk factors, and long-term health effects of hysterectomy in low and middle-income countries [1, 9]. In India, policymakers, health care providers, and researchers have sought to address reports of widespread use of hysterectomy among young women [10]. India’s National Family Health Surveys in 2015-16 and 2019-20 reported that nearly 1 in 10 women have undergone hysterectomy by age 50, ranging up to 1 in 5 in the states of Andhra Pradesh and Telangana [11, 12]. Amongst women 40-49 years, the median age at hysterectomy was 37 years, approximately a decade earlier than the age of natural menopause in India (48 years) [1, 11]. The survey found the most common self-reported reason for undergoing hysterectomy among women 15-49 years was excessive menstrual bleeding, followed by fibroids/cysts.

A growing body of evidence from high-income settings suggests that hysterectomy, particularly with oophorectomy, is a contributing factor in chronic disease in women’s mid-life and beyond [13–15]. In India, despite growing policy-level concern over high prevalence amongst young women, there is limited evidence on the long term consequences of hysterectomy on women’s health. Early hysterectomy in India, before age 45 years, renders women exposed to a considerably longer menopausal phase without oestrogen as compared to other settings, which in turn may contribute to greater risk or accelerated onset of non-communicable disease [8, 16]. This paper utilises a nationally representative survey amongst women 45 years and older to examine: (i) the prevalence of hysterectomy and correlated risk factors and (ii) associations between history of hysterectomy and women’s health status.

## Methods

### Study design and setting

This analysis is based on cross-sectional data from the first wave of the Longitudinal Aging Study of India (LASI), the first nationally representative survey of older adults in India [17]. Conducted by the International Institute of Population Studies (IIPS) and partners [18] in the year 2017-18, the survey covered 29 states and 6 union territories (excluding Sikkim). The LASI Wave 1 survey aimed to examine health status amongst India’s older population, such as the prevalence of chronic diseases including hypertension, asthma, diabetes and depression. The survey used a multi-stage, stratified area probability cluster sampling design, with three-stage sampling in rural areas and four-staged sampling in urban areas, to generate national and state-level estimates. Households were eligible if they had at least one individual over 45 years of age. In selected households, each consenting adult aged ≥45 in the household was interviewed, along with their spouse to obtain information on economic, social, and health characteristics. The survey included 72,250 participants (men and women ≥45 years and their spouses), with an individual response rate of 87.3%. We utilised data on 35,083 women ≥45 years of age.

### Variables

The survey had a specific module on women’s health that covered history of hysterectomy and the self-reported reason for undergoing the procedure. However, age at the time of procedure was not recorded. A chronic disease module included history of professional diagnosis with a chronic condition and the year of diagnosis. Diseases included were hypertension, diabetes, high cholesterol, chronic lung disease, chronic heart disease, stroke, bone/ joint diseases, and neurological/psychiatric diseases. Biomarker measures included height and weight, hand grip strength and blood pressure, along with physical examinations for lung function and visual acuity. Mobility was assessed based on self-reported difficulties expressed on a list of nine activities,^1^ categorised as none (no problems), or one or more issues (at least one issue). Depression was measured using the 10-item Center for Epidemiological Studies-Depression (CES-D) scale. Women self-reported any hospitalisation in the past 12 months.

### Statistical methods

We generated a dichotomous variable for hysterectomy status and calculated prevalence estimates with 95% confidence intervals (CIs). We present age-specific prevalence in 5-year age bands and women aged > 60 years. Missing data are reported in each table. Multivariable logistic regression was performed to identify risk factors associated with hysterectomy. We included variables reported in our previous analysis with younger women and the published literature on hysterectomy in India [9, 11, 19]. The model adjusts for demographic characteristics, including age, years of schooling (none, 1–5 years, 5–10 years and more than 10 years), place of residence (rural/ urban), marital status, number of children, caste, and tribal status (i.e. following government-issued categories for vulnerable groups: Scheduled Tribe (ST), Scheduled Caste (SC) and Other Backward Class (OBC),^2^ and other), religion (Hindu/Muslim/ Christian/Other), monthly per capita expenditure (MPCE) (categorized into five quintiles by LASI as a measure of economic status), Body Mass Index (BMI), measured using the biomarker measurements of height and weight [BMI = weight, kg/ (height, m)^2^], and classified (as per WHO guidelines) as underweight (BMI ≤18.4), normal (BMI 18.5 to 24.9), overweight (BMI 25 to 29.9), and obese (BMI ≥30), and employment history. We include a fixed effect for state, as previous analyses indicate considerable variation by state [19]. Adjusted Wald tests were used to calculate *p*-values for categorical variables with more than two categories.

We estimated adjusted odds ratios of the association between hysterectomy status and 12 conditions that have been previously reported as potential long-term effects of hysterectomy [5, 13, 14, 16, 20]. These were: (i) self-reported chronic disease: hypertension, diabetes, bone/ joint diseases, high cholesterol, chronic lung disease, chronic heart diseases, stroke and neurological/psychiatric conditions; (ii) hospitalisation in the last 12 months for any condition (iii) clinically measured blood pressure, hand grip strength and mobility [21]. All models were adjusted for demographic characteristics (age, education level, place of residence, marital status, number of children, caste, religion and socioeconomic status) and state. Associations with hypertension, diabetes, high cholesterol, chronic lung and heart disease, stroke, bone/joint disease, and mobility issues included adjustment for BMI as a potential confounder, as studies conducted in India have reported an association with hysterectomy and these conditions [22–24]. The median age at diagnosis for each morbidity was estimated from self-reported data in the survey, adjusted with survey weights.

All analyses were conducted in Stata 13 using sampling weights at the household and individual level to account for the multi-stage sampling design. Sampling weights, as recommended by the main survey report, were used to reduce bias that may arise due to the survey design, differential sampling rates, non-responses and post-stratification adjustments [18]. Although this secondary analysis of publicly available data did not involve patients directly, we will disseminate findings through previously established networks of women’s groups and researchers engaged in hysterectomy advocacy in India [25]. We report findings according to the STROBE guidelines for observational studies.

## Results

**Fig. 1:**
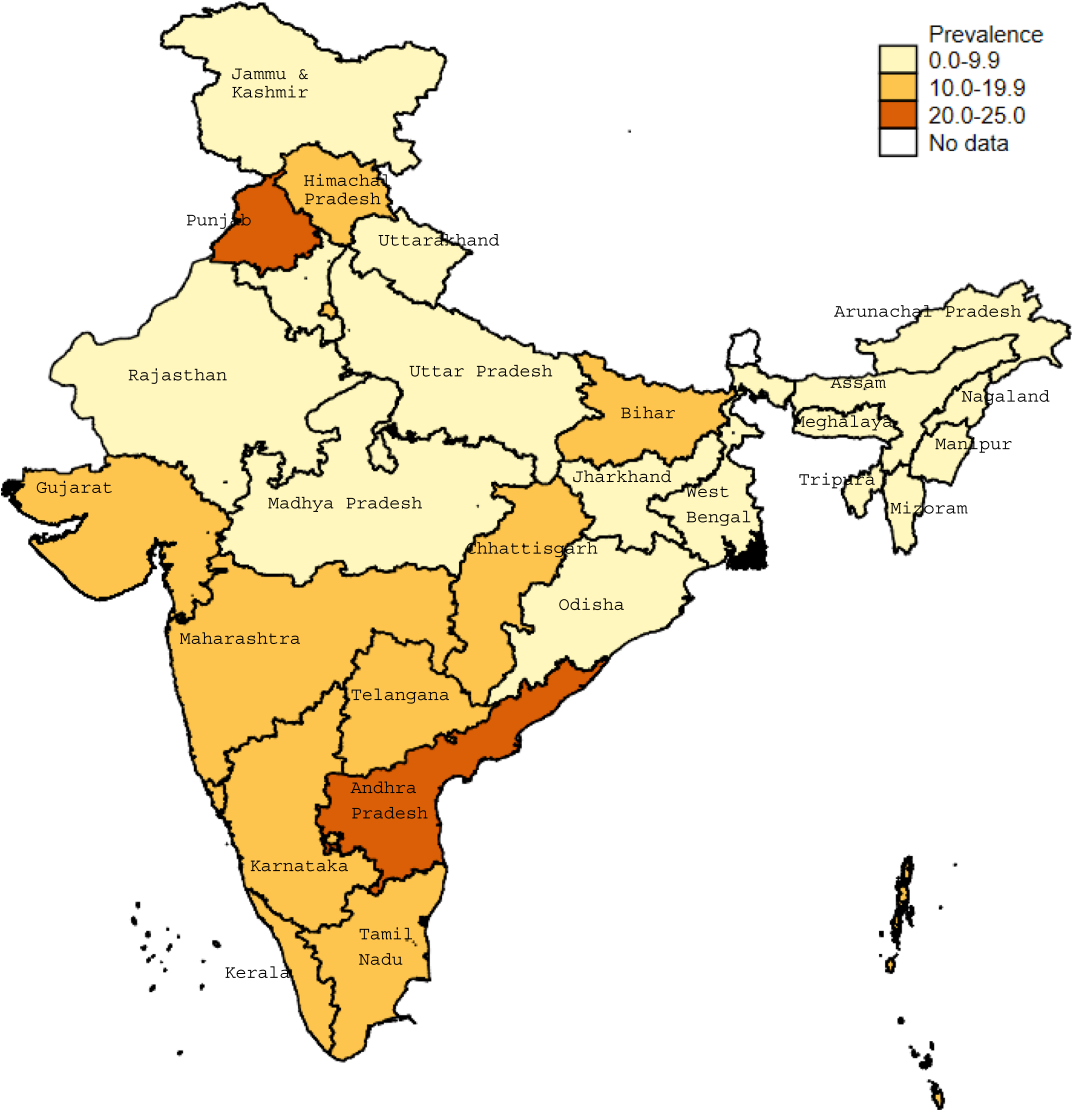
Prevalence of hysterectomy amognst women ≥45, LASI 2017-2018

The prevalence of hysterectomy among women 45 and older was 11.4%  with highest prevalance amongst women 45-59 and lower prevalence among women over 60 years (Table 1). The median age of women with hysterectomy was 57 years [IQR: 50, 65] and women without hysterectomy was 59 [IQR: 51, 68]. The leading self-reported reasons for hysterectomy were excessive menstrual bleeding/pain (33.7%), fibroids/cysts (25.5%) and uterine prolapse (17.1%) (Fig. 1), with some variation in indications across age groups. Women in ages 45-49, for example, had higher reported excessive menstrual bleeding/pain.

**Table 1:** Prevalence and self-reported reasons for hysterectomy amongst women >=45 years, LASI (2017). (Unweighted n)

	All women, age ≥ 45	Women aged 45-49	Women aged 50-54	Women aged 55-59	Women aged 60+
	***N*** = 35,083	***N*** = 7273	***N*** = 5908	***N*** = 5536	***N*** = 16,366
**Prevalence of Hysterectomy, % (95% CI)**	11.4 (10.3, 12.6)	13.8 (10.6, 17.7)	11.9 (10.5, 13.5)	13.1 (11.1, 15.5)	9.8 (8.3, 11.5)
**N**	**3648**	**762**	**678**	**660**	**1549**
Missing observations	164	44	25	25	70
**Self-reported indication for hysterectomy, %(n)**					
Excessive menstrual bleeding/ pain	33.7 (1172)	39.5 (240)	33.1 (227)	29.3 (215)	32.5 (490)
Fibroid/ cyst	25.5 (1128)	26.1 (262)	30.5 (218)	27.4 (220)	22.4 (428)
Uterine prolapse	17.2 (618)	15.0 (108)	19.9 (114)	14.2 (91)	18.5 (305)
Uterine disorders/ rupture/ injury	11.2 (329)	20.5 (64)	9.0 (67)	8.2 (67)	8.0 (131)
Reason unknown	9.3 (462)	6.7 (98)	10.2 (77)	9.5 (80)	10.5 (207)
Severe postpartum haemorrhage	4.4 (618)	3.3 (32)	4.6 (31)	3.8 (34)	5.1 (73)
Cancer	2.2 (95)	1.9 (20)	2.5 (18)	1.8 (13)	2.4 (44)
Other	0.3 (20)	0.5 (4)	0.3 (5)	0.3 (2)	0.2 (9)

Hysterectomy prevalence varied widely across Indian states (Fig. 1). In two states, Andhra Pradesh and Punjab, the prevalence of hysterectomy was higher than 20% of women ≥45 years. Prevalence was higher than 10% in Southern states, while states in Central and Northeast India reported lower prevalence. The self-reported reason for hysterectomy varied in the two highest prevalence states: almost one-half of women (46.3%) in Andhra Pradesh reported excessive menstrual bleeding compared to 19.4% in Punjab. Uterine prolapse was reported by only 5.8% of women in Andhra Pradesh compared to 23.8% in Punjab (Supp Fig. 1)**.**

Compared to women living in rural areas, urban residents were more likely to have had hysterectomy (aOR: 1.39; 1.17,1.64), as well as those with higher household economic status (highest compared to lowest quintile: aOR: 1.95; 1.44, 2.63) (Table 2). Compared to women with normal BMI, underweight women had lower odds (aOR: 0.63; 0.53,0.76) and overweight women had higher odds of history of hysterectomy (aOR: 1.31, 1.05,1.64). There was some variation by caste and religion; women from tribal communities had lower odds of hysterectomy compared to other groups. We found no evidence of an association of hysterectomy with employment history or educational attainment.

**Table 2:** Characteristics associated with hysterectomy status, women >=45 years, LASI (2017)

	Women with Hysterectomy	Women without Hysterectomy	Total	Adjusted OR^**b**^
	*N* = 3648	*N* = 31,435	*N* = 35,083^a^	(95% CI)
Age				
age 45-49	23.9 (761)	19.4 (6468)	20.0 (7273)	ref
age 50-54	16.5 (678)	15.8 (5205)	15.8 (5908)	0.92 (0.72, 1.19)
age 55-59	17.6 (660)	15.1 (4851)	15.3 (5536)	1.19 (0.92, 1.54)
age 60+	42.0 (1549)	49.8 (14747)	48.8 (16366)	0.92 (0.7, 1.19)
*p value*				0.103
Education				
Never attended school	56.9 (2011)	66.0 (19243)	64.8 (21334)	ref
Up to primary	20.9 (898)	18.1 (6369)	18.5 (7315)	0.93 (0.77, 1.14)
Middle school and higher	22.0 (739)	15.7 (5658)	16.5 (6431)	0.85 (0.56, 1.29)
*p value*				0.677
Place of Residence				
Rural	57.1 (2193)	69.0 (20254)	67.5 (22535)	ref
Urban	42.8 (1455)	30.9 (11017)	32.4 (12548)	1.32 (1.1, 1.59)
*p value*				0.003
Marital status				
Currently married	67.3 (2548)	60.9 (19421)	61.6 (22058)	ref
Never married	0.1 (16)	1.1 (345)	1.0 (364)	0.15 (0.04, 0.59)
Others ^c^	32.4 (1084)	37.9 (11505)	37.3 (12661)	0.82 (0.65, 1.03)
*p value*				0.003
Number of children ^d^				
No child	1.9 (79)	3.3 (1120)	3.2 (1206)	ref
1-2 children	32.9 (1028)	25.0 (8143)	25.8 (9185)	1.16 (0.76, 1.79)
3 or more children	64.4 (2579)	71.1 (21859)	70.1 (24402)	1.25 (0.83, 1.88)
missing observations	30	149	290	
*p value*				0.508
Caste				
Other Backward Class (OBC)	53.7 (1590)	44.1 (11453)	45.1 (13108)	ref
Scheduled Caste (SC)	16.0 (653)	19.8 (5234)	19.3 (5910)	0.81 (0.68, 0.97)
Scheduled Tribe (ST)	4.2 (306)	9.2 (5775)	8.7 (6109)	0.52 (0.39, 0.69)
Other	23.8 (977)	24.0 (7662)	24.0 (8682)	0.92 (0.74, 1.14)
missing observations	122	1147	1274	
*p value*				< 0.001
Religion				
Hindu	83.3 (2848)	81.6 (22684)	81.8 (25649)	ref
Muslim	9.4 (284)	11.6 (3945)	11.3 (4254)	0.55 (0.41, 0.73)
Christian	2.4 (243)	3.5 (3278)	3.3 (3534)	0.84 (0.62, 1.14)
Other	4.6 (273)	3.2 (1364)	3.4 (1646)	1.17 (0.84, 1.64)
*p value*				< 0.001
Monthly per capita expenditure (quintile)				
1 (lowest)	14.3 (504)	22.1 (6438)	21.1 (6976)	ref
2	16.8 (618)	21.8 (6456)	21.2 (7104)	1.24 (0.99, 1.57)
3	18.6 (710)	20.7 (6317)	20.4 (7058)	1.26 (0.98, 1.63)
4	23.7 (839)	18.9 (6128)	19.4 (7004)	1.73 (1.27, 2.35)
5	26.5 (977)	16.3 (5932)	17.6 (6941)	1.76 (1.32, 2.34)
*p value*				< 0.001
BMI category				
Underweight	9.1 (350)	19.3 (5330)	18.1 (5690)	0.63 (0.53, 0.76)
Normal	39.0 (1461)	43.1 (13839)	42.4 (15315)	ref
Overweight	28.5 (1022)	18.7 (6436)	19.8 (7467)	1.31 (1.05, 1.64)
Obese	11.7 (512)	8.2 (2740)	8.5 (3258)	1.1 (0.81, 1.5)
missing observations	303	2926	3353	
*p value*				< 0.001
Ever employed for 3 or more months				
No	43.9 (1785)	45.7 (14875)	45.6 (16736)	ref
Yes	56.0 (1863)	54.2 (16394)	54.3 (18344)	1.09 (0.9, 1.32)
missing observations	0	2	3	
*p value*				0.360

^a^Includes missing data = 164 from the hysterectomy indicator where respondents reported don’t know (*n* = 33), refused (*n* = 8) and missing observations (*n* = 123)

^b^Adjusted for state

^c^Others includes widowed, divorced, deserted, separated, live in relationship

^d^Number of living children include biological, adopted and step-children

We found evidence for an association (*p* < 0.05) between hysterectomy and four chronic conditions, compared to women without hysterectomy: hypertension (aOR: 1.53; 95% CI: 1.29, 1.81); diabetes (aOR: 1.57; 1.19, 2.06); high cholesterol (aOR: 1.44; 1.05, 1.98); and bone/joint disease (aOR: 1.48; 1.16, 1.92) (Table 3). Women with hysterectomy had higher odds of any hospitalisation in the last twelve months (aOR: 1.69; 1.36, 2.09) compared to women who have not had the procedure. Analyses stratified by wealth quintile for each outcome showed no evidence of effect modification. We found no evidence of higher odds of lung or heart disease, stroke, neurological conditions, depression, measured hypertension, hand grip strength, or mobility among women with hysterectomy. Age at diagnosis for diabetes was 10 years later, and bone/joint disease 7 years later, for women with hysterectomy compared to those without hysterectomy. There was limited difference in ages at diagnosis for hypertension and high cholesterol.

**Table 3:** Health status amongst women > 45 years, India, LASI (2017)

	Prevalence amongst all women ≥ 45 yrs	Prevalence, by hysterectomy status % (95%CI)				AOR^**a**^ (95% CI)	Median age at diagnosis [IQR], by hysterectomy status	
	% (n)	with hysterectomy		w/o hysterectomy			with hysterectomy	w/o hysterectomy
	***N*** = 35,083	***N*** = 3648	n	***N*** = 31,435	n			
**Chronic diseases**^**b**^								
Hypertension	30.9 (11,327)	43.4 (37.6, 49.3)	1550	29.4 (28.3, 30.5)	9743	1.53 (1.29, 1.81)***	45 [45, 54]	50 [40, 58]
Diabetes	12.1 (4384)	22.4 (16.0, 30.6)	670	10.9 (9.9, 11.9)	3701	1.57 (1.19, 2.06)***	60 [55, 60]	50 [38, 56]
High Cholesterol	2.2 (1337)	3.7 (2.9, 4.6)	243	2.1 (1.8, 2.4)	1083	1.44 (1.05, 1.98)**	50 [48, 55]	53 [45, 58]
Chronic Lung Disease	6.1 (1829)	5.5 (4.0, 7.6)	200	6.2 (5.4, 7.2)	1625	0.92 (0.63, 1.36)	53 [50, 53]	48 [35, 54]
Chronic Heart Disease	3.4 (1090)	3.7 (2.8, 4.8)	159	3.4 (2.6, 4.4)	924	0.90 (0.58, 1.38)	48 [26, 50]	53 [49, 60]
Stroke	1.4 (475)	1.4 (1.0, 2.1)	53	1.4 (1.3, 1.7)	420	1.28 (0.81, 2.04)	60 [48, 73]	53 [45, 60]
Measured hypertension	18.8 (6600)	21.0 (18.1, 24.2)	742	18.6 (17.6, 19.7)	5851	1.11 (0.92, 1.34)	na	na
**Strength and mobility**^**b**^								
Bone/Joint Diseases	19.0 (6162)	25.3 (20.1, 31.4)	815	18.3 (17.4, 19.4)	5335	1.48 (1.15, 1.92)***	62 [60, 62]	55 [45, 62]
Hand grip strength, Mean (SD)	17.3 (5.4)	17.5 (5.2)	3301	17.3 (5.4)	27,953	p^c^ = 0.899	na	na
Mobility issues	69.6 (23,736)	68.6 (62.8, 73.8)	2614	70.0 (68.8, 71.2)	21,079	1.14 (0.96, 1.35)	na	na
**Mental health**								
Neurological/Psychiatric	2.4 (782)	2.0 (1.5, 2.7)	98	2.5 (2.2, 2.9)	681	0.81 (0.53, 1.23)	50 [40, 50]	40 [40, 57]
Depression	29.6 (9390)	24.6 (23.5, 25.7)	1008	29.6 (28.5, 30.7)	8370	1.07 (0.87, 1.32)	na	na
**Hospitalisations**								
Hospitalisation in last year	9.2 (2286)	14.0 (11.9, 16.5)	406	8.5 (7.9, 9.2)	1872	1.69 (1.36, 2.09)***	na	na

All conditions were self-reported except measured hypertension, hand grip strength and mobility

*p* values: < 0.01***; < 0.05**

^a^ All regressions are adjusted for: age, education, wealth, caste, religion, urban/ rural, state and marital status

^b^ Additionally adjusted for BMI

^c^ Using t test for difference of means

## Discussion

This paper reports on the first population-based study of hysterectomy in India among women in mid-life and older that includes analyses of associations with women’s health status. Approximately 1 in 8 Indian women aged 45 years and above had already undergone hysterectomy, ranging up to 1 in 5 in the states of Andhra Pradesh and Punjab. The leading, self-reported reasons for hysterectomy in this study were symptoms of common gynaecological ailments, such as excessive menstrual bleeding and self-reported fibroid/cysts. We found evidence of an association between history of hysterectomy and diabetes, hypertension, bone/joint diseases, high cholesterol and hospitalisation amongst women in this survey. In addition, our results indicated a puzzling variation in the age at diagnosis for chronic disease by hysterectomy status, with no clear pattern across conditions.

While there are no comparable population-based estimates of hysterectomy in other South Asian studies, the prevalence of hysterectomy amongst women ≥45 years in India was higher than that reported in 2017 in China, where approximately 7% of women aged 45-54 had undergone hysterectomy [26]. Women living in urban areas and from higher economic strata had greater odds of reporting a hysterectomy than rural dwellers and those in poorer households. In contrast, our earlier analysis of India’s National Family Health Survey (2015-16) of women aged 15-49 found higher odds of hysterectomy amongst rural women with less education [11]. This difference across age groups may reflect changes in India’s health system over time. Expanding access to surgical procedures in rural areas, as reflected in increasing caesarean rates [27], could explain higher rates of hysterectomy amongst younger, rural women not seen in this older cohort. Similarly, lower rates in younger urban women at present, compared to the LASI cohort, could reflect increasing availability of alternative treatment options for gynaecological morbidity.

Further, the state-wise distribution of hysterectomy reflects well-established variation in health systems in Indian states. States with hysterectomy prevalence higher than 20%, such as Andhra Pradesh and Punjab, are ranked 2nd and 5th respectively in the national NITI Aayog health performance index, which includes both health outcomes and health systems capacity [28]. The higher prevalence of hysterectomy may reflect higher access to surgical infrastructure, while extremely low prevalence in others, such as in the Northeast, likely reflects inadequate infrastructure and unmet need for treatment for gynaecological ailments, including hysterectomy [29]. If we were to consider caesarean section rates as a proxy for access to surgical services, both states had caesarean section rates higher than the national average [27]. However, our previous analyses amongst women aged 15-49 years found this was not a consistent correlation; several states with similarly high caesarean section rates report low prevalence of hysterectomy [11]. There has been considerable debate in India over the role of publicly-funded health insurance schemes in incentivising hysterectomy. In this analysis, the two states with highest hysterectomy prevalence diverged regarding insurance coverage: 75% percent of households were covered by health insurance in 2015-16 in Andhra Pradesh, while only 21% were insured in Punjab [30]. Moreover, reasons for the procedure differed considerably in these two states (Supp Fig. 1), suggesting differences in the epidemiological burden of gynaecological morbidity and/or treatment offered to women. Further research on the health system drivers of hysterectomy is warranted, along with analysis of treatment pathways for gynaecological morbidity.

### Hysterectomy and women’s health

Both the LASI and NFHS surveys in India indicated the use of hysterectomy for gynaecological ailments such as excessive menstrual bleeding and cysts/fibroids, which are typically amenable to non-invasive procedures. Population-level surveys on gynaecological morbidity suggest that reproductive tract infections and menstrual disorders are common amongst reproductive-aged women in India, but barriers to seeking treatment persist in most states [31]. Qualitative research amongst low-income women in rural areas has indicated that hysterectomy was commonly used as a first or second-line procedure to treat gynaecological morbidity amongst young, rural women, due to distance, cost barriers and provider motivations [9, 32].

When hysterectomy is accompanied by removal of both ovaries (bilateral oophorectomy), the procedure surgically induces menopause, which in turn can accelerate the onset of non-communicable disease. There are no nationally representative data in India on ovarian preservation with hysterectomy; however, audits in two tertiary care hospitals found that between 38 to 59% of hysterectomies included oophorectomy [33, 34]. Reports suggest that hormone replacement therapy is not typically offered to women who undergo hysterectomy (with or without oophorectomy) in India [35]. LASI did not record history of oophorectomy, which in any case could be difficult for women to distinguish in self-reports.

Observational evidence from LASI of an association between diabetes, hypertension, bone/joint diseases, and high cholesterol amongst Indian women with hysterectomy is consistent with a growing body of evidence on the role of reproductive health events in the onset of chronic disease in later life [8]. Longitudinal data from Australia [36], the United States [6] and Taiwan [13] suggest that hysterectomy may be an contributing risk factor for specific chronic diseases. For example, an analysis of pooled data across ten observational studies in four countries indicated hysterectomy with ovarian removal at < 35 years was associated with 2.5-fold higher risk of cardiovascular disease (HRR: 2.55, 95% CI: 2.22-2.94) [16]. Recent evidence from women in Australia who underwent hysterectomy with oophorectomy before age 50 with no hormone therapy had 1.8 higher odds of all-cause mortality [36]. The physiological mechanisms for these effects are commonly linked to a decline in ovarian function and depletion of oestrogen, but more research is needed to understand the specific role of reproductive hormones in risk of chronic disease [8]. High prevalence of hysterectomy in parts of India, combined with exceptionally low median age and the potentially common use of oophorectomy, could lead to similar patterns; there is a critical need to examine the longer term effects of hysterectomy over time specific to the Indian context.

### Strengths and limitations

This paper reports on the first analysis of hysterectomy using nationally representative data amongst older women in India. While the cross-sectional survey design prevented causal inference on risk factors for hysterectomy and associations with chronic disease, it provides insight into state-level variation and identifies key indications for further study. Reverse causality and shared risk factors cannot be ruled out, particularly because LASI did not collect data on age at hysterectomy, type of hysterectomy (abdominal or laparoscopic), health status at the time and whether oophorectomy was conducted. Further, lack of age at hysterectomy limits our understanding of pathways to outcomes. We could not determine whether hysterectomy was conducted before or after diagnosis of chronic diseases—and thus cannot explain the non-consistent but substantial difference in age at diagnosis by hysterectomy status and whether hospitalization in the past year was for the surgery itself. Self-reported hysterectomy, as a major surgery, is unlikely to be subject to recall bias. However, self-reported diagnosis of conditions such as high cholesterol may have underestimated the prevalence in this population, particularly amongst women with limited access to health care.

This analysis is a critical start to understanding the role of reproductive factors on chronic disease in Indian women. There is much to be learned about the physiological and health system mechanisms, and the time to onset, for the potential link between hysterectomy and non-communicable disease. Specific details on types of gynaecological morbidity that led to hysterectomy, such as endometriosis or fibroids, can provide details required to explore alternative explanations for an association with chronic disease, such as the role of hormonal imbalance. Clinical measurement of bio-markers for non-communicable disease will be important to identify women not yet screened for risk factors due to poor access to health care. At the health system level, further research is required on the treatment pathways for gynaecological morbidity that lead to hysterectomy, viable treatment interventions in different settings, and on understanding how health service access drives inequities at the state and population sub-group level. Accordingly, longitudinal clinical, population and sociological research is required in the Indian context to understand the short- and long-term effects of hysterectomy, as well other reproductive life events, on women’s life through the life course.

## Conclusions

The associations identified in this study highlight the importance of addressing key events in the reproductive life span of women and their interlinkages with later health. Orienting service delivery towards women’s health through the life course will require a closer understanding of risk factors, including reproductive health events, and potential prevention strategies for women in specific contexts. Non-communicable diseases are the leading cause of mortality amongst Indian women [37]. The potential contribution of hysterectomy in this burden calls for critical appraisal of how the surgery is utilised, and for whom, along with feasible alternatives that protect women’s health and enhance their well-being.

## Supplementary Information

**Additional file 1: Supplementary Fig. 1.** Self-reported causes of hysterectomy in Andhra Pradesh and Punjab

## Data Availability

Data for this study are publicly available with the International Institute for Population Studies (IIPS), Mumbai upon request.

## Footnotes

1 Activities include: (i) Walking 100 yards, (ii) Sitting for 2 hours or more, (iii) Getting up from a chair after sitting for long period, (iv) Climbing one flight of stairs without resting, (v) Stooping, kneeling, or crouching, (vi) Extending arms above shoulder level (either arm), (vii) Pulling or pushing large objects, (viii) Carrying weights over 5 k, and (ix) Picking up a coin from a table.

2 Backwards classes are a collective group of castes recognised as educationally and/or socially disadvantaged by the Government of India, including: low social position in the traditional caste hierarchy; lack of general educational advancement; inadequate or no reservation in Government service; or inadequate representation in the field of trade, commerce and industry.
