# Supplementary material for: Hysterectomy and women’s health in India: evidence from a nationally representative, cross-sectional survey of older women
Source: Womens Midlife Health. 2023 Jan 6;9:1. doi: 10.1186/s40695-022-00084-9 (PMC9825041; doi:10.1186/s40695-022-00084-9)
Supplement: Supplementary file 1 — Additional file 1: Supplementary Fig. 1. Self-reported causes of hysterectomy in Andhra Pradesh and Punjab [file 40695_2022_84_MOESM1_ESM.docx]

**Supplementary Figure 1: Self-reported causes of hysterectomy in Andhra Pradesh and Punjab**
